# Ballistic trauma caused by military rifles: experimental study based on synthetic skull proxies

**DOI:** 10.1007/s12024-021-00432-7

**Published:** 2022-01-01

**Authors:** Seth C. Taylor, Benjamin Ondruschka, David C. Kieser, Niels Hammer, Matthew Lee, Gary J. Hooper, Elena Kranioti

**Affiliations:** 1 Department of Orthopaedics and Musculoskeletal Medicine, University of Otago, Christchurch, New Zealand; 2 Institute of Legal Medicine, University of Medical Center Hamburg-Eppendorf, Hamburg, Germany; 3 Department of Macroscopic and Clinical Anatomy, Medical University of Graz, Graz, Austria; 4 Department of Trauma, Orthopedic and Plastic Surgery, University Hospital of Leipzig, Leipzig, Germany; 5 Fraunhofer Institute for Machine Tools and Forming Technology, Dresden, Germany; 6 Forensic Medicine Unit, Department of Forensic Sciences, Medical School, University of Crete, Heraklion, Greece

**Keywords:** Military rifles, Gunshot wound head trauma, Synbone®, Ballistic simulations, Experiments

## Abstract

Rifles are often involved in violent deaths such as homicide and suicide. Consequently, expert knowledge and experimental forensic investigations are important to clarify the nature of ballistic trauma when applied to the human head and neurocranium. This study investigated differences in entrance wound morphology with Synbone® spheres which are described as being comparable to human flat bones. A series of ballistic experiments were conducted using two different rifle calibers (5.56 × 45 mm and 7.62 × 39 mm Full Metal Jacket (FMJ)). Synbone® spheres were used for close-range 0.3 m simulated executions as well as at 25 m and 35 m to simulate urban and military engagements. Results were compared with previously published experimental studies using similar military ammunition. In our study, entry wound morphology closely resembles real forensic cases compared to exit wound and overall shape morphology independently of the distance and the caliber. Circumferential delamination was clearly visible with full metal jacket (FMJ) rounds, yielding similar damage pattern morphology to the human crania. This study documented the presence of hydraulic burst or shock in all ten rounds from all three distances. Krönlein shots were also observed in some cases. Synbone® spheres constitute an acceptable synthetic surrogate for ballistic experiments. The present study offers new initial data on the behavior of Synbone® proxies in ballistic testing of military ammunitions; FMJ gunshot injuries to the human head, for distances that have not previously been published, suggesting that efficient tests can take place under these conditions. Further research on experimental ballistics with a larger number of controlled factors and multiple repetitions is recommended to verify the results of this pilot study before applied in forensic simulations.

## Introduction

In recent years there has been a global increase in gun violence, with roughly 500 individuals a day dying from firearm violence alone [1]. Forensic investigators need to be able to provide detailed knowledge on wound morphology, potential shot distances, and firearm calibers, both as individual factors and in combination to legal authorities as part of the initial investigation, or later as an expert witness to a court of law. Experimental ballistics and incident simulations have been broadly used as means to understand the effect of high energy impact on human proxies. Trauma simulations including ballistic testing have used both animal [2, 3] and synthetic surrogates [4], spheres [5, 6] and anatomically correct head proxies with variable results [7]. Whilst synthetic proxies for long bones appear problematic [4, 8, 9] it has been suggested that polyurethane spheres present efficient proxies for crania [6].

The anatomical region of the head has gained particular interest with the pioneer work of Thali and colleagues [10–12] who introduced the “skin-skull-brain” model followed by the more recent use of synthetic analogues in an effort to study the reaction of the cranium and brain in a variety of situations involving ballistic impacts [6, 13, 14]. Thali et al*.* [11] produced a “skin-skull-brain” model which was found to be a good approximation of the human head and exhibited realistic injuries for a range of ammunition including Full Metal Jacket (FMJ). These results are supported by a more recent study on handgun executions using a range of calibers from 0.22 to 0.45 [13].

When it comes to military weapons a few scarce experiments have been conducted. Thali et al. [11] used high velocity military rifles (7.62 × 51 mm and 7.62 × 39 mm) from a distance of 10 m and reported realistic cranial and brain damage without giving specific details on the corresponding trauma patterns. Smith and colleagues [6] employed 7.62 × 51 mm NATO FMJ from a distance of 2 m and noted that “The spheres shot with modern rifles … compare favourably with published examples of modern cranial gunshot trauma” citing several published forensic reports. A more recent study by Mahoney and colleagues [14] tested the effect of assault rifles (7.62 × 39 mm) on synthetic proxies in distances of 50-100 m and noted that “the produced fractures were too comminuted when compared to recent military injuries”.

Considering the limited information provided in the literature on the synthetic proxies’ behavior following ballistic impacts of high velocity rifle rounds, this current pilot study aims to expand on the topic by testing on a similar cranial proxy across different shooting distances that are common in urban warfare. More specifically this paper uses the two most common rounds found in recent conflict situations, the 7.62 × 39 mm and 5.56 × 45 mm rounds [15], shot from distances of 0.3, 25 and 35 m in an effort to provide more data on the mechanical behavior of the synthetic proxies (Synbone® spheres filled with ballistic gelatin) shot by distances that have not been tested so far. In addition, this research introduces the use of treated pig skin as human skin proxy in order to create a more realistic head model.

## Materials

### Synbone® spheres

Ten Synbone® spheres were used in this study. Synbone® products are advertised primarily as proxies of human bone for use within surgical training but above have also been used with ballistic studies. Synbone® has a range of spheres available, some of which are produced with a rubber skin designed to simulate the periosteum. The spheres are formed of two hemispheres glued together, which simulates a single suture running continuously around the sphere’s diameter and are coated with a rubber skin. At the apex of the hemispheres there is a 4 cm hole to replicate the presence of the foramen magnum, this allows for the spheres to be filled with ballistic gelatin to simulate the crania with brain present. Spheres with a diameter of 190 mm and a wall thickness of 7 mm were used in this study. This thickness falls within the range of human frontal and occipital bones [16, 17].

### Ballistic gelatin

The spheres were filled with a solution of 360 g of Fluka type 3 porcine gelatin mixed with 3.2 L of water. This gelatin is commonly used as a proxy for human brain tissue [11].The spheres were then chilled at 10% at 4 ºC for 24 h to set the gelatin and shot within ten minutes of being removed from the chiller.

### Sus scrofa skin

Two of the ten spheres were covered with *Sus scrofa* (pig) skin which was sourced and produced by a local per European standards of butchery. The pig skin was chosen and shot to verify if pig skin is a viable natural proxy to human skin.

### Ammunition

This study used FMJ ammunition of the calibers 7.62 × 39 mm and 5.56 × 45 mm [18, 19]. These rounds are classified as high velocity rounds [20, 21] (see Table 1 for ammunition specifications).

**Table 1:** Specification of the ammunition used within this study

Calibre	Bullet Type	Bullet Weight	Muzzle Velocity	Muzzle Energy
5.56 × 45mm^a^	FMJ	4.0 g	920 m/s	1700 J
7.62 × 39mm^b^	FMJ	8.0 g	738 m/s	2188 J

^a^After Army ammunition data sheets [18]

^b^After Wolf ammunition [19]

## Methods

### Sus scrofa skin

The purchased skin was processed by removing excess internal fat and tissue so that it reached a thickness of 3-4 mm. This thickness is consistent with the thickness of the skin found on the human cranium [22]. The skin was cut to size, wrapped around the sphere, and was sewn in place using a butcher’s trussing needle and butcher’s twine to form a tight-fitting cover over the sphere leaving the foramen magnum exposed.

### Experimental setting

The prepared spheres were placed upon a cork ring on top of a table with a single strand of tape securing them both to the table, following the method shown in Taylor and Kranioti [13]. The top of the spheres measured 112 cm off the ground, this simulates a man of average height (178 cm) kneeling on the ground [23]. The spheres wrapped in pig skin were positioned to ensure that the sides facing towards and away from the shooter were a smooth section of skin without suture lines.

The spheres were divided into two groups to provide intra- and inter-caliber comparison and were shot offhand by the same shooter. Some variation in angle is implied. These were shot from three distances, each intended to simulate real-world close and intermediate ranges. Muzzle velocity was not measured due to the short distances, with velocities assumed to be within manufactures parameters. Shots from 0.3 m were modelled on executions where the victim is kneeling in front of the shooter [24]. The two longer distances (25 m and 35 m) were used to simulate more intermediate ranges that may occur in close range combat situations in urban fighting [6]. Table 2 outlines the breakdown of which spheres were shot with which caliber and from which distance.

**Table 2:** The conditions under which each Synbone® sphere was shot, outlining whether the sphere had a pig skin cover, the calibre used, and the distance between the sphere and the muzzle

Sphere	Sphere Covering	Calibre	Distance
1	Pig Skin + rubber skin	5.56 × 45 mm	0.3 m
2	rubber skin	5.56 × 45 mm	0.3 m
3	Pig Skin + rubber skin	7.62 × 39 mm	0.3 m
4	rubber skin	7.62 × 39 mm	0.3 m
5	rubber skin	7.62 × 39 mm	25 m
6	rubber skin	5.56 × 45 mm	25 m
7	rubber skin	7.62 × 39 mm	25 m
8	rubber skin	5.56 × 45 mm	25 m
9	rubber skin	5.56 × 45 mm	35 m
10	rubber skin	7.62 × 39 mm	35 m

### Data acquisition

The experimental procedure was filmed using a Cannon EOS 1200D camera with a 50 mm lens, with the camera placed 0.3 m from the spheres at a 45º angle to them. After the shooting sequence the spheres and ballistic gelatin were physically examined to identify information such as the placement of the entrance and exit “wounds”, circumferential delamination (CD), fracture patterns, the appearance of Hydraulic Burst (HB) [25] and Krönlein shot pattern was scored for each case.

Following the suggestion of Mahoney and colleagues [14] and in an effort to produce comparable results a Likert-type scale was used to assess entrance, exit wound and general appearance of the produced fracture pattern (where 4 = exactly like a real injury, 3 = a lot like a real injury, 2 = a bit like a real injury and 1 = nothing like a real injury).

## Results

### Krönlein shot

A complete evisceration of the brain simulant (called a Krönlein shot) [26–28] was observed with a 0.3 m execution recreation firing a 7.62 × 39 mm round in both shot trials, as displayed in Table 3. However, the smaller 5.56 × 45 mm ammunition with a lighter 4.0 g bullet and less muzzle energy did not cause a Krönlein shot at 0.3 m in both trials, irrespective of using the sphere with or without a skin layer.

**Table 3:** The presence and absence of circumferential delamination (CD), hydraulic burst (HB), and Krönlein shots and the shooting conditions under which they occurred

Sphere	Calibre	Distance	Sphere Covering	CD	HB	Krönlein Shot
1	5.56 × 45 mm	0.3 m	Pig Skin	Yes	Yes	No
2	5.56 × 45 mm	0.3 m	Rubber skin	Yes	Yes	No
3	7.62 × 39 mm	0.3 m	Pig Skin	Yes	Yes	Yes
4	7.62 × 39 mm	0.3 m	Rubber skin	Yes	Yes	Yes
5	7.62 × 39 mm	25 m	Rubber skin	Yes	Yes	No
6	5.56 × 45 mm	25 m	Rubber skin	Yes	Yes	No
7	7.62 × 39 mm	25 m	Rubber skin	Yes	Yes	No
8	5.56 × 45 mm	25 m	Rubber skin	Yes	Yes	No
9	5.56 × 45 mm	35 m	Rubber skin	Yes	Yes	Yes
10	7.62 × 39 mm	35 m	Rubber skin	Yes	Yes	No

### Circumferential delamination (CD)

**Fig. 1:**
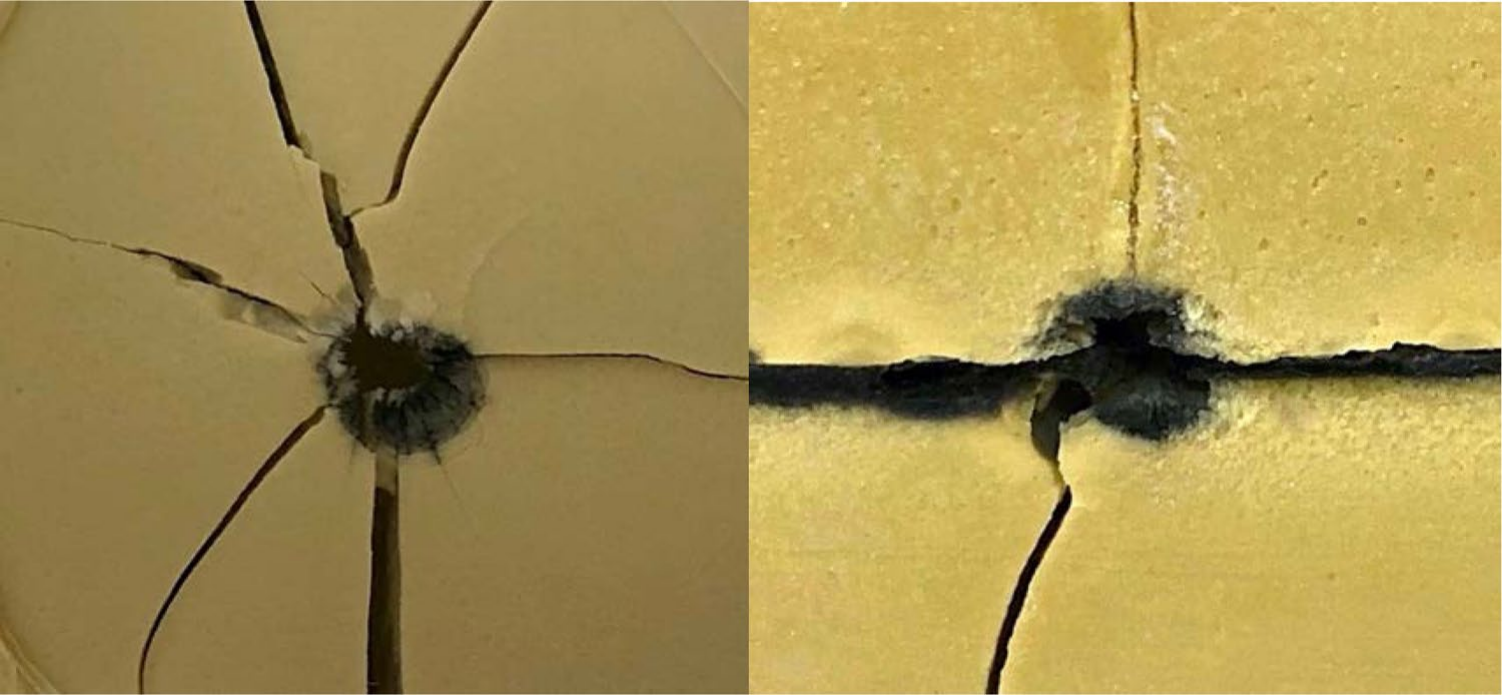
Circumferential delamination from a 7.62 × 39 mm FMJ ammunition shot (left) and 5.56 × 45 mm FMJ ammunition shot (right) to a Synbone® sphere at 25 m distance

All rounds exhibited circumferential delamination patterns to varying extent (Fig. 1), as summarized in Table 3. Circumferential delamination occurs due to the crushing of the bone around the rim of the entrance wound due to the hardness of the FMJ round as it moves through bone or in this case the polyurethane of the Synbone® sphere [13, 29].

### Hydraulic burst effect (HB)

This study documented the presence of hydraulic burst or shock in all ten rounds from all three distances (0.3 m, 25 m, and 35 m), as summarized in Table 3. The hydraulic burst is the result of the bullet passing through fluid-filled hollow organs (brain is considered as such) a medium that allows significant build-up of pressure that can burst the organ [25].

In our study, entry wound morphology more closely resembles real forensic cases. Exit wound morphology appears nothing like the real injury when only Synbone spheres are shot. However, resemblance improves when pig skin covering is used in addition. In distances less than 10 m Krönlein shots are more frequently described when 7.62 × 39 and 7.62 × 51 mm are used. Moreover, in > 50 m distances, the appearance of Krönlein shots does not follow a particular pattern of association.

## Discussion

This study aimed to expand upon the work done by Taylor and Kranioti [13], Smith et al. [6] Thali et al. [10, 11], and Mahoney et al. [7, 14] on testing whether Synbone® spheres are good proxies for human crania in ballistic studies and to record different injury patterns observed at different distances. It was found that the entry points of the ammunition into the spheres exhibit similar morphological patterns to real forensic cases when shot from a distance less than 35 m while exit wounds were larger than documented forensic cases. The radiating fractures seen in the present study were also consistent with those seen when Synbone® spheres were shot with FMJ pistol rounds [13] and as expected in real cases. Contrary to this, entry wound patterns seemed little consistent with real cases in shots from distances of 50-100 m according to one study [14]. This seems highly dependent of the distance, although other factors such as wind velocity and angle of the shot cannot be discarded. Synbone® sphere thickness was 1 mm smaller in the last study [14] but again this small discrepancy seems highly unlikely to be responsible for the diverse injury pattern observed in these rounds.

Circumferential delamination (CD) is another featured observed in the current study. It has been argued that CD is a feature indicative of FMJ rounds in previous research [29]. The hardness of the FMJ rounds comparative to the bone it moves through, crushes the bone or proxy around the rim of the entrance wound, leading to the appearance of CD. This has been observed consistently in a previous experimental study using handguns and FMJ rounds [13]. The present study employed two FMJ military rounds fired from distances of 0.3 to 35 m and it was observed that all Synbone® spheres exhibited CD at the entrance wound across all three shooting distances and caliber sizes (See Table 3). This also agrees with the study of Smith et al. [6] that described CD in a sphere shot with a 7.62 × 51 mm caliber from a distance of 2 m. Unfortunately studies by Thali and colleagues [10] at 10 m and Mahoney and colleagues at 50-100 m [14] do not describe any CD in the entry wounds, however, it is not clear if this is because the authors did not include this feature in their research protocol or because they feature was absent.

**Fig. 2:**
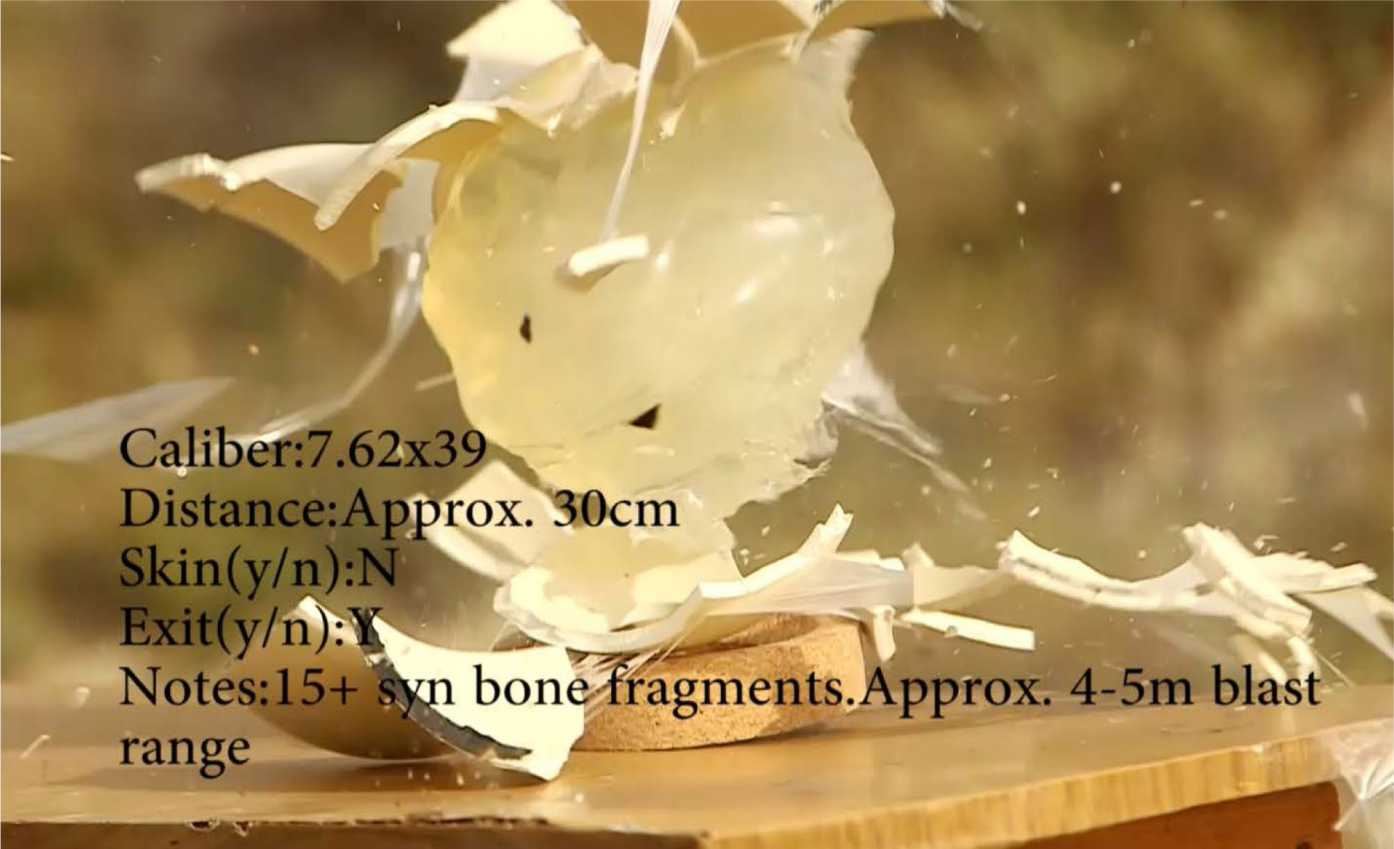
Hydraulic burst of a Synbone® sphere at 30 cm with a 7.62 × 39 mm

Also witnessed in this study was the hydraulic burst effect (Fig. 2) [20, 25]. The human head with the brain present is considered to be a hollow organ (brain tissue being classified as a liquid). High velocity rounds cause an immense amount of hydraulic pressure inside a hollow organ, and in the presence of liquid (which cannot be compressed) it will cause the organ, such as a human head, to rupture [25]. Synbone® spheres filled with gelatin have been shown to replicate this effect better than other proxies, such as bovine scapulae [30], as not all of these can recreate the hollow organ nature of the cranium like the spheres can. Di Maio [20] stresses that “a high-velocity bullet fired through an empty skull produces small entrance and exit holes with no fractures” while “same missile fired through a skull containing brain causes extensive fracturing and bursting injuries”. The head proxies used in our experiment fulfilled the requirement of “hollow liquid-filled” organs, thus, all shots across the three shooting distances produced this effect. This was not the case in the handgun experimental study from 0.3 m where only three of the six pistol calibers produced this effect [13]. This is probably due to lower energy produced in the smaller rounds. The hydraulic burst effect was not mentioned in other studies thus, no assumptions can be made on additional factors that may affect its appearance in the experiments using high velocity military ammunition.

Krönlein shots [28] were also observed in the 7.62 × 39 mm and 7.62 × 51 mm rounds and distances < 10 m while it exhibits an inconsistent pattern in 7.62 × 39 mm rounds shot from distances of 50-100 m. Krönlein shots occurred at 0.3 m for both 7.62 × 39 mm spheres with and without skin covering, which is likely related to the amount of energy of 2188 J produced by the round compared to 1700 J produced with 5.56 × 45 mm. Interestingly, Krönlein shot occurred at 35 m with the 5.56 × 45 mm. This shot could have happened due to the muzzle velocity of the round at 35 m [18] which is still past the threshold of the velocities needed for a Krönlein shot to occur (< 800 m/s) [31]. Yet, this cannot not explain why the 5.56 × 45 mm did not cause Krönlein shots at the closer distances in our study. Discrepancies in the appearance of a Krönlein shot are also noted in the study of Mahoney and colleagues [14] at 50-100 m for with 7.62 × 39 mm rounds. Besides the longer distances in the latter study, Synbone® thickness is 1 mm thinner compared to ours but even considering these two factors the random appearance of Krönlein shots in the Mahoney study (2019) cannot be explained due to lacking information such as firing angle, wind velocity and other parameters that can affect wound morphology in high velocity military ammunition. Tangential hits, for example, may provoke a lift of the cranial vault while the brain remains undamaged [25]. These observations most certainly pose the basis for further research on experimental ballistics with a larger number of controlled factors and multiple repetitions.

The advantage of Synbone® spheres is that they are easy to obtain and pose no ethical limitations on researchers, which is in contrast to both human and animal tissues when used for this purpose. They do not come though without limitations. Whilst studies have shown that Synbone® exhibits a satisfactory reaction to impact as a cranial proxy [10–12] and can exhibit realistic reaction to ballistic injuries as demonstrated in the current study (e.g., hydraulic burst, Krönlein shot) there are still questions as to its suitability as a proxy for long bones [7–9]. Also, when ballistic testing on Synbone ® spheres used 7.62 × 39 mm ammunition at 50 m and 100 m the resulting injury patterns were considered to be “too comminuted and fragmented” when compared to contemporary military injuries encountered by participating physicians [12].

The study itself exhibits several additional limitations. Due to the financial constraints of purchasing multiple Synbone® spheres the sample size was limited to five spheres per caliber for this pilot study. This limited the shot distances to three different distances which represent extreme versions (executions and longer shots) of realistic cases. We have chosen to include 0.3 m so that results can be comparable to our previous study on handguns fired from the same distance [13] and the remaining distances in order to fill the gap encountered in the literature complementing other experimental studies on military ammunition [6, 11, 14]. This drawback needs to be addressed in future studies by expanding the number of spheres used at each distance and the calibers used. Regardless, the present study offers new initial data on the behavior of Synbone® proxies in ballistic testing of military ammunitions for distances that have not previously been published, suggesting that efficient tests can take place under these conditions.

Taking into account the results of this and previous studies [6, 11, 13, 14] there is evidence that Synbone® spheres filled with ballistic gelatin with or without extra skin substitute can be efficient head proxies to simulate gunshot inflicted trauma using a variety of guns and ammunition. Yet, due to the number of uncontrolled factors and limited repetitions of the so far reported experiments, definite conclusions on wound ballistics cannot be drawn with confidence. Instead, it seems safer to use artificial head proxies to simulate specific conditions of a shooting incidents where multiple factors can be controlled according to the scene evidence. A good example of this principle is the experimental study by Mahoney and colleagues [7] that simulated two military combat inflicted injuries with the use of helmet for which a number of factors such as engagement distance, bullet manufacturer, batch and propellant load being controlled. Repetitive experiments (3 per case) gave however a range of bullet trajectories indicating the possible influence of more uncontrolled factors in the experiments. This leaves room for more experimentation in future studies.

## Conclusion

Macroscopically, Synbone® spheres perform well as a proxy to human flat bones for high velocity rounds in terms of circumferential delamination and the phenomenon of hydraulic burst effect. All FMJ rounds replicated realistic injuries on head proxies employed in this study at 0.3 m, 25 m and 35 m distance as opposed to experiments of larger distances reported by others. This can be attributed to a variety of uncontrolled factors in combination with the distance. While synthetic head proxies are proven efficient proxies to simulate gunshot inflicted trauma using a variety of guns and ammunition, it is proposed to be used to test scenarios of documented trauma for which a larger number of factors is controlled and a sufficient number of experimental repetitions is possible so that results can be safely corroborated. This pilot study implies further research in experimental ballistics is warranted.

## Key Points

1. Rifles are often involved in violent deaths such as homicide and suicide.
2. Expert knowledge and experimental forensic investigations are important to clarify the nature of ballistic trauma when applied to the human head and neurocranium.
3. Synbone® spheres were used for close-range 0.3 m simulated executions as well as at 25 m and 35 m to simulate urban and military engagements.
4. Entry wound morphology closely resembles real forensic cases compared to exit wound and overall shape morphology independently of the distance and the caliber.
5. Synbone® spheres constitute an acceptable synthetic surrogate for ballistic experiments.

## Author contibutions

Taylor: main author as research was undertaken in course with a PhD program. Lee: main body and editing. Kieser, Hammer, Hooper and Ondruschka: supervision and editing. Kranioti: study design, supervision, interpretation of the data and approval of the final manuscript.
